# Plant Terpenoids as the Promising Source of Cholinesterase Inhibitors for Anti-AD Therapy

**DOI:** 10.3390/biology11020307

**Published:** 2022-02-14

**Authors:** Shereen Lai Shi Min, Sook Yee Liew, Nelson Jeng Yeou Chear, Bey Hing Goh, Wen-Nee Tan, Kooi Yeong Khaw

**Affiliations:** 1 School of Pharmacy, Monash University Malaysia, Bandar Sunway 47500, Malaysia; shereenlai98@gmail.com; 2 Chemistry Division, Centre for Foundation Studies in Science, University of Malaya, Kuala Lumpur 50603, Malaysia; joeyliew5382@um.edu.my; 3 Centre for Natural Products Research and Drug Discovery (CENAR), University of Malaya, Kuala Lumpur 50603, Malaysia; 4 Centre for Drug Research, Universiti Sains Malaysia, Minden, Penang 11800, Malaysia; nelsonchear@usm.my; 5 Department of Medicinal Chemistry, College of Pharmacy, University of Florida, Gainesville, FL 32610, USA; 6 Biofunctional Molecule Exploratory (BMEX) Research Group, School of Pharmacy, Monash University Malaysia, Bandar Sunway, Subang Jaya 47500, Malaysia; Goh.bey.hing@monash.edu; 7 College of Pharmaceutical Sciences, Zhejiang University, Hangzhou 310058, China; 8 Chemistry Section, School of Distance Education, Universiti Sains Malaysia, Penang 11800, Malaysia

**Keywords:** terpene, nor-abietanoid, serratene, cholinesterase, Alzheimer’s disease

## Abstract

**Simple Summary:**

Plant-derived terpenes have been a research interest in the recent years, as they are believed to possess the ability to function as a cholinesterase inhibitor. As the deficit of cholinergic activity is one of the factors that causes cognitive impairment in Alzheimer’s disease patients, it serves as a great therapeutic target. It has been found that various terpenoids, such as diterpenoids, triterpenoids and sesquiterpenoids, do have the ability to inhibit cholinesterase activity, and their chemical structures do play a role in this. As terpenoids possess anti-cholinesterase properties, it is encouraged to have future research on drug discovery and development in treating Alzheimer’s disease.

**Abstract:**

Plant-derived terpenes are the prolific source of modern drugs such as taxol, chloroquine and artemisinin, which are widely used to treat cancer and malaria infections. There are research interests in recent years on terpene-derived metabolites (diterpenes, triterpenes and sesquiterpenes), which are believed to serve as excellent cholinesterase inhibitors. As cholinesterase inhibitors are the current treatment for Alzheimer’s disease, terpene-derived metabolites will have the potential to be involved in the future drug development for Alzheimer’s disease. Hence, a bibliographic search was conducted by using the keywords “terpene”, “cholinesterase” and “Alzheimer’s disease”, along with cross-referencing from 2011 to 2020, to provide an overview of natural terpenes with potential anticholinesterase properties. This review focuses on the extraction, chemical structures and anti-cholinesterase mechanisms of terpenes, which support and encourage future research on drug discovery and development in treating Alzheimer’s disease.

## 1. Introduction

**Figure 1:**
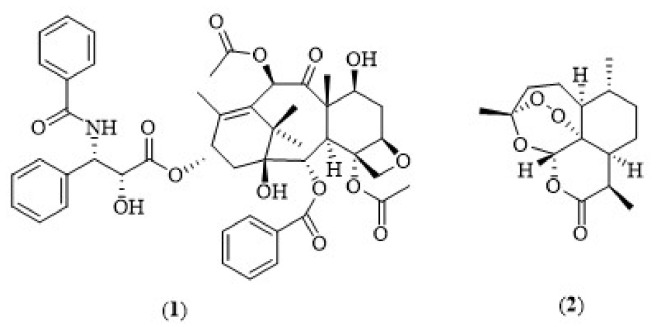
Chemical structures of terpene-derived drugs.

Terpenes are the largest and most diverse class of secondary metabolites produced by plants [1]. They are simple hydrocarbons that possess multiple isoprene units. Meanwhile, terpenoids are terpene derivatives containing different functional groups. Terpenoids can be classified into the following: one isoprene unit (hemiterpenoids), two isoprene units (monoterpenoids), three isoprene units (sesquiterpenoids), four isoprene units (diterpenoids), five isoprene units (sesterterpenoids), six isoprene units (triterpenoids) and eight isoprene units (tetraterpenoids) according to the number of isoprene units or carbon atoms that form the parent terpene [2,3]. Terpenoids have been widely used for the treatment of many diseases due to their broad range of biological activities, such as antimicrobial, anticancer, anti-hyperlipidemic, anti-hyperglycemic, anti-inflammatory, antioxidant, anti-parasitic, immunomodulatory and anti-cholinesterasic activities. For instance, Taxol^®^ (paclitaxel) (**1**) (Figure 1) is one of the mostly used and clinically well-established terpene-based chemotherapy drugs [4]. On the contrary, artemisinin (**2**) is another terpene-based drug derived from sweet wormwood (*Artemisia annua*) which has been widely used as an anti-malarial medication [5,6]. Among the various biological activities exhibited by terpenoids, anti-cholinesterase activity has gained the attention of researchers. Cholinergic hypothesis is the accepted approach to explain Alzheimer’s disease (AD) pathology, which asserts the deficit of acetylcholine (ACh) level in the brains of AD patients. Other than acetylcholinesterase (AChE), butyrylcholinesterase (BuChE) is also a point of interest, as it is an alternative target for AD treatment due to the surge in its enzymatic activity found in the late stage of AD. In this review, we provide a comprehensive summary of terpene-derived cholinesterase inhibitors focusing on their extraction, chemical structures, anti-cholinesterase actions and structure–activity relationships (SARs).

## 2. Cholinergic Hypothesis in Pathogenesis and Treatment of AD

**Figure 2:**
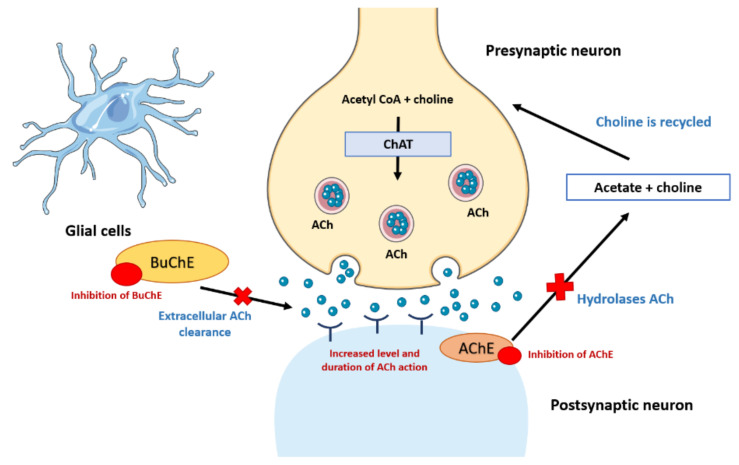
Cholinergic hypothesis in pathogenesis and treatment of AD.

**Figure 3:**
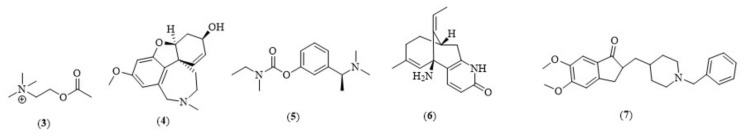
Cholinesterase neurotransmitter and cholinesterase inhibitors (**3**–**7**).

Severe loss of cholinergic neurons in the nucleus basalis and associated areas that form the cholinergic forebrain area and affected cerebral cortices are common pathological changes among AD patients. Figure 2 showed an overview of cholinesterase hypothesis in the pathogenesis of AD. These factors cause a great reduction (>90%) in the activity of choline acetyltransferase, a crucial receptor for ACh (Figure 3) [7]. The deficit of cholinergic activities causes various cognitive impairments, including memory loss. Moreover, the elevation in AChE level, an enzyme that is responsible in terminating the physiological role of acetylcholine at cholinergic synapses, is another hallmark among AD patients [7]. When such changes occur, the level of brain BuChE, which is an enzyme that regulates extracellular ACh level, is found to be elevated. Hence, BuChE can be considered as an alternative drug target for AD treatment as well. These findings lead to the hypothesis that cholinergic augmentation might improve cognition in AD patients by restoring ACh level through the inhibition of cholinesterase enzymatic activity [8]. The main function of cholinesterase inhibitors is to inhibit the activity of cholinesterase enzymes (AChE and BuChE). Subsequently, this helps to increases the ACh level in the brain, which leads to the improvement of the central cholinergic function. To date, the cholinesterase enzyme inhibitor remains as the standard approach for the symptomatic treatment of AD [9,10]. Some examples of potent cholinesterase inhibitors, such as galanthamine (**4**), rivastigmine (semi-synthesized) (**5**), huperzine A (**6**) and donepezil (**7**) (synthetic drug), are shown in Figure 3.

## 3. Diterpenoids

Diterpenoids (diterpenes) are a class of terpenes with a C20 carbon skeleton comprising four isoprene units. They are gaining scientific interest owing to their vast chemical diversity and reputed medicinal properties [11,12,13]. In the last decade, several diterpenoids derivatives from plants have been reported as promising cholinesterase inhibitors targeted at AChE and BuChE. For instance, *Caryopteris mongolica* and *Lycopodiastrum casuarinoides* [14,15].

### 3.1. Abietane Diterpenoids

**Figure 4:**
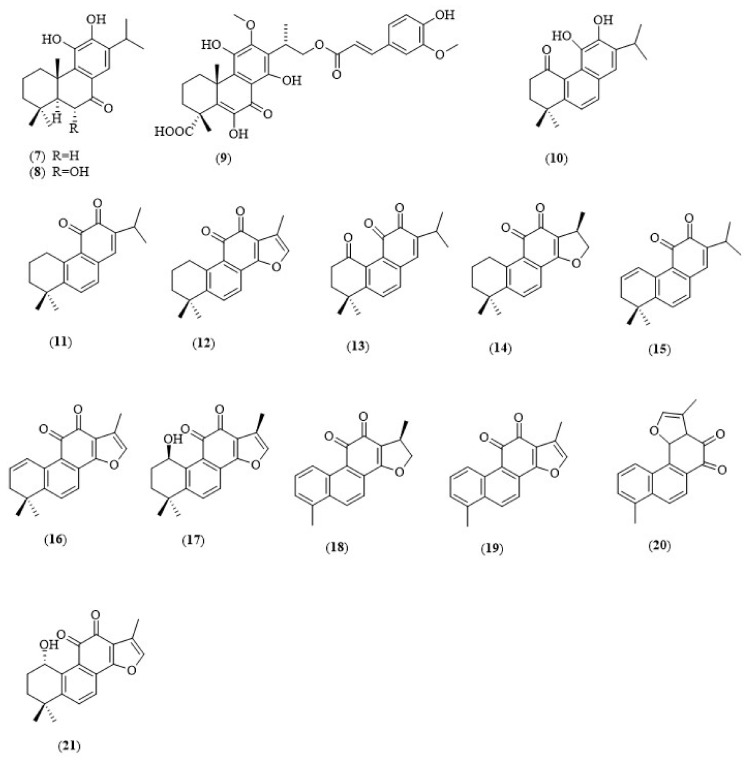
Chemical structures of diterpenoids as cholinesterase inhibitors (**7**–**21**).

Studies have shown that abietane diterpenoids possessed potent inhibitory effect toward cholinesterases. For instance, abietane diterpenoids that were isolated by Murata et al. from *Caryopteris mongolica* (Figure 4), a medicinal plant which is traditionally used in the treatment of aches, rheumatism and edema, are proven to have potent inhibitory effect against AChE and BuChE. Murata et al. extracted the aerial parts of *C. mongolica* with a mixture of polar solvents, acetone:H_2_O (8:2), which was subjected to a partition between water and diethyl ether. A preparative high-performance liquid chromatograph (HPLC) was performed to purify the compounds [14]. Human erythrocytes were used as a source of AChE and horse serum was used as a source of BuChE. The enzymatic results have shown that the abietane diterpenoids, 12-O-demethylcrptojaponol (**7**) and 6α-hydroxydemethylcryptojaponol (**8**) inhibited both human erythrocyte AChE and horse serum BuChE enzymes with IC_50_ values in micromolar range, in which compound (**7**) was a dual cholinesterase inhibitor (AChE IC_50_ = 50.8 μM, BuChE IC_50_ = 70.1 μM), while compound (**8**) was a BuChE selective inhibitor (AChE IC_50_ = 19.2 μM, BuChE IC_50_ = 7.7 μM) (Table 1). The substitution of a hydroxyl group at the C-6 position of compound (**8**) is well correlated with its higher cholinesterase inhibitory activity (10-fold) compared to compound (**7**) [14]. Furthermore, Liu et al. reported a series of potent abietane diterpenoids from *Lycopodiastrum casuarinoides*, a traditional Chinese herb to treat pain. Extraction of the air-dried aerial part of *L. casuarinoides* was conducted with 75% EtOH, followed by silica-gel chromatography to obtain nine compounds, including the isolation of a new abietane diterpenoid, lycocasuarinone A (**9**). Cholinesterase inhibitory activities were performed by using modified Ellman’s method, and compound (**9**) was active against AChE with an IC_50_ value of 26.8 μM [15]. Interestingly, compound (**9**) was found to have an additional feruloyl moiety compared to lycopodabietane A [15], suggesting the inhibitory effect against AChE. Furthermore, feruloyl group possessed antioxidant and anti-inflammatory effects, which can prevent neuroinflammation. It also enhances novel-object recognition performance and averted memory loss [16]. Additionally, feruloyl group inhibits the formation of amyloid-β-peptide (Aβ) oligomers, which is significant in the pathogenesis of AD [17,18,19].

### 3.2. Tanshinones

Tanshinones, a class of abietane diterpenes, can be found in the roots of *Salvia* and *Perovskia* species. These compounds are proven to possess potent neuroprotective and anticholinesterase activities with high permeability crossing the blood–brain barrier (BBB) [20,21]. Senol et al. isolated twelve nor-abietanoids from *Perovskia atriplicifolia* Benth and *Salvia glutinosa* L., which were active against cholinesterase enzymes. Among the tested compounds, 15,16-dihydrotanshinone (**18**) has the most potent inhibitory effect against AChE (65.17% inhibition), while having an affinity for BuChE at the same time (94.88% inhibition, IC_50_ = 1.71 μM). It is suggested that the structural difference of this compound on the carbon-15 position of the furan ring might result in its inhibitory effect toward the cholinesterases. Oppositely, miltirone (**11**) was found to be the most potent BuChE inhibitor (98.36% inhibition), followed by cryptotanshinone (**14**), 1,2-didehydromiltirone (**15**) and 1β-OH-cryptotanshinone (**17**). Compound (**11**) did not show any inhibitory effects toward AChE, and this might be due to the presence of the isopropyl group in its structure, instead of furan rings [22]. Molecular docking studies showed that the most potent dual cholinesterase inhibitor, 15,16-dihydrotanshinone (**18**), interacts with both AChE and BuChE enzymes primarily through pi–pi stacking (and hydrophobic) and hydrogen bonds, respectively. In a docking study, compound (**18**) forms a pi–pi stack and hydrophobic bonds with two key amino acids, TYR334 and PHE330, situated in the gorge of the AChE active site, blocking acetylcholine access to the catalytic triad and inhibiting the enzyme. The study revealed that (**18**) forms hydrophobic bonds with three critical residues: TRP82, LEU286 and VAL288 within the binding pocket of BuCHE. In addition, through hydrogen bonding, the carbonyl group of (**18**) interacts with HIS438 from the catalytic triad. 

In another study, four selected tanshinone congeners—tanshinone IIa (**12**), cryptotanshinone (**14**), 15,16-dihydrotanshinone (**18**) and tanshinone I (**19**)—were evaluated for their cognition-enhancing effects in a mice passive-avoidance task model [23]. In this study, a single administration of the selected tanshinones alone significantly increased the step-through latencies (retention trial) compared to the vehicle-treated control group, thus indicating that these compounds enhanced learning and memory. Further, these compounds were found to reverse the cognition deficit induced by scopolamine (1 mg/kg, i.p.), an anticholinergic drug, in a dose-dependent manner. Scopolamine is a tropane alkaloid that acts as a muscarinic antagonist, blocking the activity of muscarinic acetylcholine receptors. Both experiments (with and without scopolamine pretreatment) revealed that compounds (**12**) and (**18**) (2 and 4 mg/kg, p.o.) have a greater cognition-enhancing effect than compounds (**14**) and (**19**) (10 and 20 mg/kg, p.o.), and are more effective than the known AChE inhibitor, tacrine (10 mg/kg, i.p.). All the tanshinones studied had strong acetylcholinesterase inhibitory effects on mouse homogenate (ex vivo model), thus further substantiating their action as AChE inhibitors in improving mice’s cognitive performance in the passive-avoidance task model. These compounds, on the other hand, also enhanced the mice’s cognitive performance in the diazepam-induced cognition-impairment model. Diazepam is a benzodiazepine-type anxiolytic which often causes side effects, such as cognitive impairment, related to their ability to over-activate nitric oxide synthase (NOS) in the brain. Thus, this study also partly suggests that tanshinones (**12**,**14**,**18** and **19**) may enhance the cognitive performance in the diazepam model via the suppression of brain NO synthase (as a NOS inhibitor). Hence, tanshinones could be developed as potential leads for AD treatment, as they are able to inhibit cholinesterase (AChE and BuChE) and nitric oxide–mediated neuroinflammation, which are the key pathological events of AD.

**Table 1:** Diterpenoids isolated from plants with their IC_50_ values of cholinesterase inhibitory activities.

Species	Plant Parts	Extraction Method	Types of Compound	Chemical Constituent	Cholinesterase Inhibition (µM) (A: AChE, B: BuChE)	References
*Caryopteris mongolica*	Aerial part	Extract with acetone water (4:1), quenched with diethyl ether and water, diethyl ether fraction was passed through HP20 column	Abietane diterpenoid	12-*O*-demethylcryptojaponol (7)	50.8 (A) 70.1 (B)	[14]
				6α-hydroxydemethylcryptojaponol (8)	12.3 (A) 7.7 (B)	
*Lycopodiastrum* *casuarinoides*	Aerial part	Extract with 75% ethanol, partitioned with EtOAc and 3% tartaric acid, column chromatography	Abietane diterpenoid	Lycocasuarinone A (9)	26.8 (A)	[15]
*Perovskia atriplicifolia* *Salvia glutinosa*	Root	Sonicate with hexane, column chromatography	Nor-abietanoid	Arucadiol (10)	4.0 (B)	[22]
				Miltirone (11)	0.90 μg/mL (B)	
				Tanshinone IIa (12)	2.79 μg/mL (B)	
				1-oksomiltirone (13)	5.06 μg/mL (B)	
				Cryptotanshinone (14)	1.15 μg/mL (B)	
				1,2-didehydromiltirone (15)	1.12 μg/mL (B)	
				1,2-didehydrotanshinone IIa (16)	5.98 μg/mL (B)	
				1β-OH-cryptotanshinone (17)	1.21 μg/mL (B)	
				15,16-dihydrotanshinone (18)	1.71 μg/mL (B)	
				Tanshinone I (19)	11.24 μg/mL (B)	
				Isotanshinone II (20)	9.16 μg/mL (B)	
				1(*S*)-OH-tanshinone IIa (21)	5.71 μg/mL (B)	

## 4. Triterpenoids

Triterpenoids are a large and structurally diverse group of terpenoids consisting of 30-carbons based on six isoprene units. Triterpenoids are biosynthesized from an acyclic C30 hydrocarbon—squalene or oxidoqualene [24]—and mostly appear as either alcohols, aldehydes or carboxylic acids in plants. Several triterpenoid derivatives, such as lanostane triterpenoids, steroids, lupine derivatives and phenylpyranotriterpenoid (dichapteralins A), have been reported as potent cholinesterase inhibitors [25].

### 4.1. Serratene-Type Triterpenoids

Serratene-type triterpenoids are a group of pentacyclic triterpenoids with a seven-membered ring and seven tertiary methyl groups. They are compose of a double bond between C-14 and C-15 and oxygen functionalities at both C-3 and C-21 positions [26]. These triterpenoids are well-known for their promising biological activities, especially their anticholinesterasic, cytotoxic and chemo-preventive activities [27]. In the study conducted by Nyugen et al. on *Lycopodiella cernua*, twelve serratene-type triterpenoids were identified and evaluated for their cholinesterase inhibitory activities. *L. cernua* is a traditional Chinese herb used to treat rheumatism, hepatitis and other illnesses. Air-dried whole plants of *L. cernua* were powdered and extracted twice with methanol under reflux. Repeated column chromatography led to the isolation of these twelve compounds. Among them, four serratene-type triterpenoids were active against AChE (IC_50_ < 30 μM), in which 3β,21α-diacetoxyserratan-14β-ol (**24**) was the most potent AChE inhibitor, followed by 3β,21β,29-trihydroxyserrat-14-en-3β-yl p-dihydrocoumarate (**25**), 3β,14α,15α,21β-tetrahydroxyserratan-24-oic acid-3β-yl-(4′-methoxy-5′-hydroxybenzoate) (**23**) and 21β-hydroxyserrat-14-en-3,16-dione (**22**), with IC_50_ values in the submicromolar range (0.91 to 10.67 μM). For BuChE inhibition, compound (**25**) was the strongest BuChE inhibitor, with an IC_50_ value of 0.42 μM (Table 2). Notably, the BuChE inhibitory activity of compound (**25**) was 3-fold greater than the positive control—berberine (IC_50_, 1.09 μM). The enzyme kinetic activities of the most active compounds, (**24**) and (**25**), were determined, and compound (**24**) was found to be a mixed-type AChE inhibitor, with a Ki value of 2.49 μM, while compound (**25**) was a dual inhibitor with different modes of inhibition against AChE and BuChE. Compound (**25**) shown mixed-type inhibition toward AChE (Ki = 7.97) and competitive inhibition toward BuChE (Ki = 0.99) [25].

### 4.2. Cucurbitane-Type Diterpenoids

Cucurbitane-type triterpenoids are tetracyclic triterpenoids that are mainly found in Cucurbitaceae species. The key feature of cucurbitane triterpenoids is their 19-(10→9β)-abeo-10α-lanost-5-ene skeleton [28]. *Cirtrullus colocynthis*, also known as desert gourd and bitter apple, is known as a rich source of cucurbitane terpenoids. It has been used as traditional Uygur medicine for the treatment of indigestion, astriction, paralysis, phlegm plug, arthritis, epilepsy, tumors and diabetes [29,30]. Liu et al. reported four new cucurbitane triterpenoids from *C. colocynthis*, *colocynthenins* A–D, and evaluated for their AChE inhibitory activities. Air-dried powdered *C. colocynthis* fruits were macerated by using methanol at room temperature and then subjected to repeated column chromatography for isolation of the compounds. Among the isolated compounds, colocynthenin A (**27**) and C (**28**) demonstrated potent AChE inhibition activity with IC_50_ values of 2.6 and 3.1 μM. Meanwhile, colocynthenin B and D showed negligible inhibition at the highest tested dose at 10 μM [29]. Structure–activities relationships (SARs) studies revealed that compound (**27**) possessed an additional unprecedented lactone ring (A′) at C-2 and C-11 positions of cucurbitacin E, and this is favorable for AChE inhibition. The AChE inhibitory potential of compound (**28**) is greatly enhanced by the addition of a cyano group at C-1 position of cucurbitacin E.

### 4.3. Limonoids

Limonoids are a group of tetranotriterpenoids. They are highly oxygenated triterpenes that are biosynthesized from the acetate–mevalonate pathway mostly found in the *Citrus* genus. These compounds occur naturally in other plant families, such as Rutaceae and Meliaceae [31,32]. In the study conducted by Dzoyem et al., three structurally related limonoids—trichilia lactone D5 (**29**), rohituka (**30**) and dregeanin DM4 (**31**)—were isolated from the seeds of *Trichilia welwitschii* with moderate AChE inhibitory effect [33]. Powdered air-dried seeds and leaves of *T. welwitschii* were extracted separately with a mixture of CH_2_Cl_2_:MeOH (1:1) at room temperature. Silica-gel column chromatography was performed to purify these limonoids. These limonoids possessed concentration-dependent inhibitory activities toward AChE. Among the tested limonoids, compound (**29**) was the most active AChE inhibitor, with an IC_50_ value of 19.13 μM, followed by (**30**), with IC_50_ = 34.15 μM, and (**31**), with IC_50_ = 45.69 μM. However, these compounds are less likely to follow the Lipinski Rule of Five, as their molecular weights are generally exceed 500 Da (between 601 and 715 Da).

### 4.4. Lanostane Triterpenoids

*Ganoderma lucidum*, which is locally known as Lingzhi in China or Reishi in Japan, is a well-known Traditional Chinese Medicine (TCM) [34]. Polysaccharides, steroids and triterpenoids—particularly, lanostane triterpenoids—were the major constituents of *G. lucidum.* Wei and colleagues reported the AChE inhibitory activity of forty-five lanostane triterpenoid derivatives from the whole plant of *G. lucidum*. Ethanolic extracts of the fruiting bodies of *G. lucidum* were extracted with chloroform and subsequently subjected to compound purification by chromatography. Results have shown that twelve triterpenoids were active against AChE, with IC_50_ values < 200 μM. Among them, two compounds, namely ganolucidic acid E (**32**) and 11β-hydroxy-3,7-dioxo-5α-lanosta-8,24(E)-dien-26-oic acid (**33**), were found to be active (IC_50_ < 50 μM) against AChE, with the IC_50_ values of 10.8 and 13.8 μM, respectively (Table 2). Structure–activity relationship (SAR) studies of twelve active *G. lucidum* terpenoids suggested that C30 ganoderic acid skeleton is favorable for AChE inhibition and that the C-17 side chain may serve as the key feature for the inhibitory effect. The SARs finding was further supported by molecular docking studies in which the two most active AChE inhibitors—(**32**) and (**33**)—possess an α,β-unsaturated carboxyl group (25-COOH) at C-17 side chain of ganoderic acid. This feature allows (**32**) and (**33**) to interact with the amino acids of AChE including LEU-138 and VAL-132, respectively [35].

### 4.5. Friedelanes

Friedelanes are pentacyclic triterpenes that are mainly found in the Celastraceae family and several other families, such as Malpighiaceae [36]. A study conducted by Liu et al. reported three novel norfriedelane triterpenoids isolated from the branches and roots of *Malpighia emarginata* with potent AChE inhibitory activity. Norfriedelin A (**44**) and norfriedelin B (**45**) were active against AChE with IC_50_ values of 10.3 and 28.7 μM, respectively. Compound (**44**) performed this way owing to its 3-fold greater cholinesterase inhibitory activity than (**45**). It might be due to the presence of α-oxo-β-lactone group in compound (**44**) that makes it more favorable to AChE, as compared to the keto-lactone group, which is present in compound (**45**) [37].

### 4.6. Other Triterpenoids

**Figure 5:**
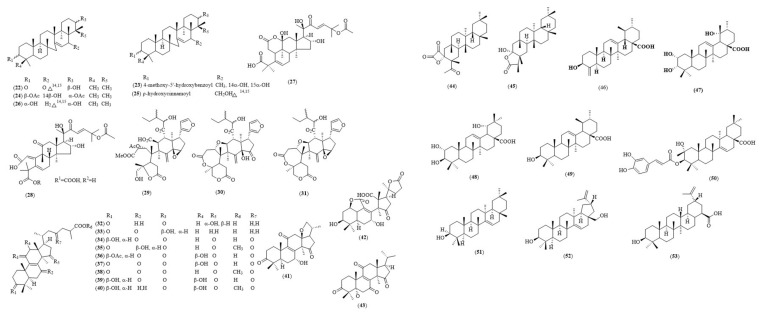
Chemical structures of triterpenoids as cholinesterase inhibitors (**22–53**).

*Patrinia scabiosaefolia* is a Chinese medicine herb traditionally used to treat initial stages of edema, appendicitis, endometriosis and inflammation. Air-dried and powdered whole plant of *P. scabiosaefolia* was extracted with ethanol, followed by silica-gel column chromatography to obtain 3β-hydroxy-24-nor-urs-4(23)-12-dien-28-oic acid (**46**). It was reported that (**46**) exhibited cholinesterase enzyme inhibitory activity with a IC_50_ value of 10.0 μM [38]. Similarly, three moderate anti-AChE triterpenes—euscaphic acid (**47**), arjunic acid (**48**) and ursolic acid (**49**)—were isolated from the leaves of *Callicarpa maingayi* K. and G. Among the isolated triterpenes, compound (**49**) showed the greatest AChE inhibitory activity, with an IC_50_ value of 21.5 μM, followed by compound (**47**) (35.9 μM) and compound (**48**) (37.5 μM). Based on the SAR study, compound (**49**) possessed the least number (without) of hydroxyl groups at both C-2 and C-19 positions, as compared to compounds (**47**) and (**48**)**.** This indicates that the presence of hydroxyl groups at these two positions weakens the AChE inhibition [39]. In another study conducted by Nargis et al., four anticholinesterse triterpenoids, namely 2β-hydroxy-3α-*O*-coffeoyltaraxar-14-en-28-oic acid (**53**), taraxerol (**51**), botulin (**52**) and betulinic acid (**53**), were isolated from the bark of *Garcinia homboniana*. Two *Garcinia* triterpenoids exhibited dual cholinesterase (AChE and BuChE) inhibition, with 2-hydroxy-3-*O*-caffeoyltaraxar-14-en-28-oic acid (**50**) being the most potent dual inhibitor (AChE IC_50_ = 13.5 μM, BuChE IC_50_ = 10.6 μM), followed by compound (**53**) (AChE IC_50_ = 24.2 μM, BuChE IC_50_ = 19.1 μM) [40]. Another two compounds, (**51**) and (**52**), were found to be either selective BuChE or AChE inhibitors with the IC_50_ values of 17.8 and 28.5 μM, respectively. Molecular docking analysis indicated that compound (**50**) interacted with both catalytic and peripheral binding sites of AChE through the formation of three hydrogen bonding, each with key amino acids—Tyr 334, His 440 and Ser 200. Figure 5 shows the examples of selected plant-derived triterpenoids.

**Table 2:** Triterpenoids isolated from plants with their IC_50_ values of cholinesterase inhibitory activities.

Species	Plant Parts	Extraction Method	Types of Compound	Chemical Constituent	Cholinesterase Inhibition (µM) (A: AChE, B: BuChE)	References
*Lycopodiella cernua*	Whole plant	Reflux with methanol, partitioned with hexane and EtOH, EtOH fraction subjected to column chromatography	Serratene	21β-hydroxyserrat-14-en-3,16-dione (**22**)	10.67 (A)	[25]
				3β,14α,15α,21β-tetrahydroxyserratan-24-oic acid-3β-yl-(4′-methoxy-5′-hydroxybenzoate) (**23**)	9.98 (A)	
				3β,21α-diacetoxyserratan-14β-ol (**24**)	0.91 (A)	
				3β,21β,29-trihydroxyserrat-14-en-3β-yl p-dihydrocoumarate (**25**)	1.69 (A) 0.42 (B)	
				serrat-14-en-3α,21β-diol (**26**)	1.37 (B)	
*Citrullus colocynthis*	Fruits	Extract with methanol, fractionated, column chromatography	Cucurbitane	Colocynthenin A (**27**)	2.6 (A)	[29]
				Colocynthenin C (**28**)	3.1 (A)	
*Trichilia welwitschii*	Seeds	Extract with dichloromethane methanol, flash chromatography	Limonoid	Trichilia lactone D5 (**31**)	28.55 (A)	[33]
				Rohituka (**30**)	57.5 (A)	
				Dregeanin DM4 (**29**)	78.37 (A)	
*Ganoderma lucidum*	Fruiting body	Extract with ethanol, fractionated and column chromatography	Lanostane	Ganolucidic acid E (**32**)	13.8 (A)	[34]
				11β-hydroxy-3,7-dioxo-5α-lanosta-8,24(E)-dien-26-oic acid (**33**)	10.8 (A)	
				Ganoderic Am1 (**34**)	183 (A)	
				Methyl ganoderate C (**35**)	148 (A)	
				Ganodernoid C1 (**36**)	142 (A)	
				12β-hydroxyganodernic F (**37**)	102 (A)	
				Methyl ganoderate E (**38**)	45.8 (A)	
				Ganoderic acid C6 (**39**)	147.5 (A)	
				Methyl Ganoderic acid C6 (**40**)	145.2 (A)	
				Gaodernoid B2 (**41**)	102.4 (A)	
				Ganoderlactone G (**42**)	130.5 (A)	
				Ganodernoid A (**43**)	149.0 (A)	
*Malpighia emarginata*	Branches and roots	Extract with acetone, fractionated, column chromatography	Norfriedelane	Norfriedelin A (**44**)	10.3 (A)	[37]
				Norfriedelin B (**45**)	28.7 (A)	
*Patrinia scabiosaefolia*	Whole plant	Extract with ethanol, partitioned with ethyl acetate, column chromatography	Triterpenoid	3β-hydroxy-24-nor-urs-4(23)-12-dien-28-oic acid (**46**)	10.1 (A)	[38]
*Callicarpa maingayi*	Leaves	Extract with methanol, fractionated, column chromatography	Triterpenoid	Euscaphic acid (**47**)	35.9 (A)	[39]
				Arjunic acid (**48**)	37.5 (A)	
				Ursolic acid (**49**)	21.5 (A)	
*Garcinia hombroniana*	Barks	Sequential extraction, column chromatography	Triterpenoid	2-hydroxy-3-O-caffeoyltaraxar-14-en-28-oic acid (**50**)	13.5 (A) 10.6 (B)	[40]
				Taraxerol (**51**)	17.8 (B)	
				Betulin (**52**)	28.5 (A)	
				Betulinic acid (**53**)	24.2 (A) 19.1 (B)	

## 5. Sesquiterpenoids

**Figure 6:**
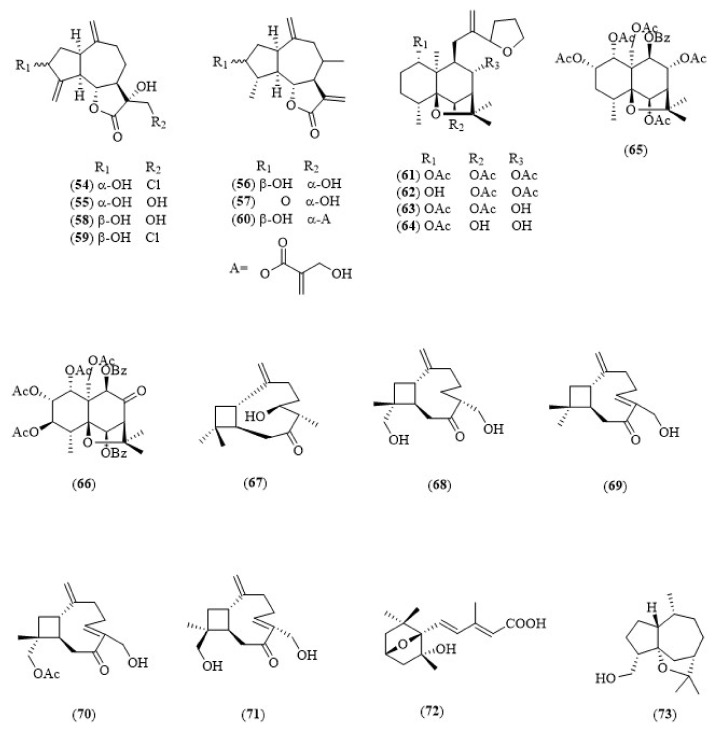
Chemical structures of sesquiterpenoids as cholinesterase inhibitors (**54–73**).

Sesquiterpenoids are C15-terpenoids with three isoprene units that occur naturally as hydrocarbons or in oxygenated forms, which include lactones, alcohols, acids, aldehydes and ketones [41]. They are the most diverse group of terpenoids, owing to a wide variety of skeletons, including linear, monocyclic, bicyclic and tricyclic frameworks [42]. Figure 6 shows the anticholinesterase sesquiterpene derivatives from plants (**54**–**73**).

### 5.1. Sesquiterpene Lactones

Hegazy et al. reported seven sesquiterpene lactones from *Cynara cornigera* (wild artichoke) owing to cholinesterase inhibition activity. The plant is traditionally used for the treatment of choleretic, diuretic, hepatoprotective and spasmolytic effects. Methanol (80%) was used for extraction, followed by extensive fractionation and purification to obtain seven sesquiterpene lactones. The results showed that a new chlorinated sesquiterpene lactone, cornigeraline A (**54**), was the most active sesquiterpene lactone with an IC_50_ value of 20.5 μM (Table 3). Notably, the AChE inhibitory effect of (**54**) was 3-fold greater than its epimer, 13-chlorosolstitialine (**59**). Compound (**54**), owing to two hydrophobic moieties located in the sesquiterpene nucleus, which is close to C-10 and between C-1 and C-3 positions, stands a good chance to pass through BBB, which makes it suitable for the management of AD [43].

### 5.2. Sesquiterpene with Agarofuran Skeletons

Sesquiterpenoids with dihydro-β-agarofuran skeletons are unique to the family of Celastraceae with a wide range of biological activities, including anti-HIV, immunosuppressive, insecticidal and cytotoxic activities. The seeds were separated and extracted with methanol. Silica gel chromatography and purification was performed to isolate the compounds. Six sesquiterpenoids with agarofuran (epoxyeudesmane) skeletons (**61**–**66**) were identified from the seeds and aerial parts of *Maytenus disticha.* The results showed that these compounds have weak but selective AChE inhibition activities at micromolar range (ranging 122–738 μM) [44].

### 5.3. Caryophyllene-Type Terpenoids

The aerial part of *Pulicaria vulgaris* is used to treat intestinal disorders, flu and inflammation. Five caryophyllene-type terpenoids, namely pulicaryenne A (**67**), (1S,6R,9S,11R)-13,14-dihydroxycaryophyll-2(15)-en-7-one (**68**), (5Z)-14-hydroxycaryophyllen-7-one (**69**), (1S,5Z,9R)-12-acetoxy-14-hydroxycaryophylla-2(15),5-dien-7-one (**70**) and (1S,5Z,9R)-12,14-dihydoxycaryophylla-2(15),5-dien-7-one (**71**), were reported for their AChE inhibitory activity. Cholinesterase inhibitory activity showed that compounds (**71**) and (**68**) have greater inhibitory potential toward AChE compared to compounds (**67**)**,** (**69**) and (**70**) at a micromolar range between 25 to 200 μM. The differences in the AChE inhibitory activities among these analogues can be explained through substitution positions and the configuration of the stereogenic centers within the caryophyllene basic skeleton. For instance, compound (**71**) exhibited a stronger AChE inhibitory effect than compound (**68**). The finding could be explained by the absence of the asymmetric center C-6 in compound (**71**) and its replacement by the endocylic double bond Δ^5,6^ through the inversion of the configuration at C-11 [45].

### 5.4. Other Sesquiterpenes

Liu et al. reported a new sesquiterpene acid, megatigma-7,9-diene-1,4-epoxy-2-hydroxy-10-carboxylic acid (**72**), from *Lycopodiastrum casuarinoides*. The compound has dual cholinesterase inhibition properties, which are used for pain treatment. Results have shown that compound (**72**) is a dual inhibitor (AChE IC_50_ = 9.3 μM; BuChE IC_50_ = 9.4 μM) [15]. On the other hand, artemisinin (**2**) and qinanol A (**73**) exhibited mild-to-moderate AChE inhibition activities (IC_50_ > 20 μM) [46,47].

**Table 3:** Summary of sesquiterpenoids isolated from different plants with their IC_50_ values of cholinesterase inhibitory activities.

Species	Plant Parts	Extraction Methods	Types of Compounds	Chemical Constituents	Cholinesterase Inhibition (µM) (A: AChE, B: BuChE)	References
*Cynara cornigera*	Aerial parts	Extract with methanol, column chromatography	Sesquiterpene lactone	Cornigeraline A (**54**)	20.5 (A)	[41]
				Sibthorpine (**55**)	35.8 (A)	
				3-hydroxy-grosheimin (**56**)	30.5 (A)	
				Grosheimin (**57**)	61.8 (A)	
				Solstitalin A (**58**)	25.7 (A)	
				13-chlorosolstitialine (**59**)	62.1 (A)	
				Cyanaropicrin (**60**)	31.3 (A)	
*Maytenus disticha*	Seeds	Extract with methanol, fractionated, chloroform fraction, further column chromatography	Agarofuran	1α,6β,8α-triacetoxy-9β-furoyloxy-β-agarofuran (**61**)	248 (A)	[44]
				1α-hydroxy-6β,8α-diacetoxy-9β-furoyloxy-β-agarofuran (**62**)	738 (A)	
				1α,6β-diacetoxy-8α-hydroxy-9β-furoyloxy-β-agarofuran (**63**)	161 (A)	
				1α-acetoxy-6β,8α-dihydroxy-9β-furoyloxy-β-agarofuran (**64**)	312 (A)	
				1α,2α,6β,8α,15-pentaacetoxy-9β-benzoyloxy-β-agarofuran (**65**)	122 (A)	
				1α-acetoxy-6β,9β-difuroyloxy-4β-hydroxy-β-agarofuran (**66**)	738 (A)	
*Pulicaria vulgaris*	Aerial part	Extract with acetone–water (1:1), fractionated, column chromatography	Caryophyllene Sesquiterpene	Pulicaryenne A (**67**)	214.85 (A)	[45]
				(1S,6R,9S,11R)-13,14-dihdroxycaryophyll-2(15)-en-7-one (**68**)	39.97 (A)	
				(5Z)-14-hydroxycaryophyllen-7-one (**69**)	108.26 (A)	
				(1S,5Z,9R)-12-acetoxy-14-hydroxycaryophylla-2(15),5-dien-7-one (**70**)	101.22 (A)	
				(1S,5Z,9R)-12,14-dihydroxycaryophylla-2(15),5-dien-7-one (**71**)	25.78 (A)	
*Lycopodiastrum casuarinoides*			Sesquiterpene acid	Megatigma-7, 9-diene-1,4-epoxy-2-hydroxy-10-carboxylic acid (**72**)	9.49 (A) 9.34 (B)	[15]
*Artemisia annua*	Leaves	Extract with ethanol, column chromatography, Sephadex LH-20	Sesquiterpene lactone	Artemisinin (**2**)	104 (A)	[46]
*Aquilaria sinensis*	Woods	Diethyl ether extract, column chromatography	5,11-epoxyguaiane	Qinanol A (**73**)	100.7 (A)	[47]

## 6. Conclusions

This review summarized the anticholinesterase activities of plant-derived terpenoids which were reported from 2011 to 2020. Although several cholinesterase-active terpene derivatives have been identified, none of them are being subjected to further preclinical (pharmacokinetic, pharmacodynamic, toxicity and CNS mechanisms) and clinical evaluations. One of the main limitations of transitioning the lead compounds into preclinical and clinical studies is to produce the desired compounds on a larger scale. Because plant-derived terpenes are generally low in extraction yield and complex in structure, they are difficult to synthesize in the laboratory. A recent review revealed that ginseng extract and its active metabolites showed beneficial roles in the pathogenesis of AD [48]. Hence, continuous research efforts and methodology inventions are needed to develop new and effective cholinesterase inhibitors from plant-derived terpenoids.

## Data Availability

Not applicable.
